# Dandy-Walker Syndrome Associated with Scoliosis: Clinical Presentation, Preoperative Assessment, and Treatment

**DOI:** 10.1155/2020/8874819

**Published:** 2020-08-17

**Authors:** Athanasios I. Tsirikos, Sarah J. Wordie

**Affiliations:** Scottish National Spine Deformity Centre, Royal Hospital for Sick Children, Edinburgh, UK

## Abstract

Dandy-Walker syndrome (DWS) affects the posterior cranial fossa resulting in characteristic dysmorphic facial and body features. Scoliosis is not typically reported as an extracranial manifestation of this condition. We present a 12-year-old female patient who developed a right thoracic scoliosis measuring 60° with increased lumbar lordosis. Scoliosis correction was indicated to alleviate back pain, improve cosmesis, and prevent respiratory complications. A multidisciplinary preoperative assessment included cardiac, respiratory, anaesthetic, and neurology reviews. She underwent a posterior spinal fusion from T2 to L3 with pedicle hook/screw and rod instrumentation and a combination of locally harvested autologous and allograft bone. This resulted in excellent deformity correction and a balanced spine in the coronal and sagittal planes. The patient made an uneventful recovery and returned gradually to her normal level of activities. She was monitored in clinic until she completed her growth (4 years after surgery); the satisfactory surgical outcome was maintained at follow-up and was associated with high patient satisfaction. Scoliosis can occur in children with DWS with resemblance to adolescent idiopathic scoliosis in regard to type of coronal deformity, age at presentation, surgical techniques, and postoperative recovery. Early identification of scoliosis in patients with DWS can allow preoperative planning and prompt surgical management in order to reduce the risk of significant morbidity which can occur if the scoliosis is allowed to deteriorate. Excellent deformity correction can be achieved and maintained beyond skeletal maturity in order to improve physical appearance, as well as preserve level of function and quality of life.

## 1. Introduction

Dandy-Walker Syndrome (DWS) comprises a spectrum of congenital intracranial abnormalities, predominantly affecting the posterior fossa. Even though DWS is a rare condition, it is the most common posterior cranial fossa malformation, which usually occurs sporadically [1]. The severity of clinical symptoms depends on the presence of a range of brain anomalies such as a dilated cyst in the 4th ventricle, enlargement of the posterior fossa, and absence of the cerebellar vermis. It is well established that patients with DWS have associated extra- and intracranial congenital abnormalities, developmental delay, and hydrocephalus [2–4].

We present a patient with DWS who developed a severe right thoracic scoliosis and underwent a posterior spinal arthrodesis. We describe the patient's preoperative presentation and postoperative outcome through to skeletal maturity, 4 years after surgery. We are aware of only one other report describing the association of spinal deformity and DWS [5].

## 2. Case Presentation

A British Caucasian female patient aged 12 years and 9 months presented to our institution with a right thoracic scoliosis, which was first noted at the age of 12 years and 5 months. She was diagnosed with DWS on the basis of her craniofacial dysmorphism and confirmed on a magnetic resonance imaging (MRI) scan obtained at age 2 years. The patient was born prematurely at 27 weeks and was ventilated for the first 12 weeks of life followed by another 2 months of oxygen support, leaving her with chronic neonatal lung disease. She was slow to achieve developmental milestones and walked independently at age 3 years. At age 5 years and 6 months, she underwent cardiac surgery to close a patent ductus arteriosus (PDA). The patient suffered from chronic constipation and dysphagia but was otherwise medically well. She also had mild coordination difficulties affecting her hands and learning problems but attended mainstream school. There was no family history of neurological/syndromic conditions or scoliosis.

At presentation to our clinic, she reported a 6-month history of increasing thoracic back pain, causing sleep disturbance. She was premenarchal, with height 147.2 cm, arm span 148 cm, body weight 32.1 kg, and BMI 14.8. On clinical examination, the patient was slimly built with normal physical characteristics for her age. She had a severe right thoracic scoliosis, which was rotated to the right and produced a right-sided rib prominence on the posterior chest wall, adjacent to the convexity of the curve. There was also thoracic translocation with listing of the trunk to the right and mild elevation of the right shoulder. There was waistline asymmetry with prominence of the left side of the pelvis, which was level. She had normal thoracic kyphosis and increased distal lumbar lordosis with anterior pelvic tilt. There was no evidence of leg-length discrepancy. There were no abnormalities to the skin or subcutaneous tissues overlying the spine, and the patient did not report any subjective neurological deficits. Neurological examination confirmed normal muscle power, sensation, and brisk tendon reflexes in the upper and lower limbs. Mild clonus was elicited bilaterally. She had normal gait and balance. Spinal movements were pain-free but straight leg raise was restricted due to significant bilateral hamstring and gastrocnemius tightness. There was no tenderness on palpation along the spine and paraspinal muscles. The patient had mild ligamentous laxity.

Radiographs of the spine taken at the patient's first assessment in clinic showed a right thoracic scoliosis extending from T6 to L2 and measuring 53°. There were 12 thoracic and 5 lumbar vertebrae with no evidence of congenital vertebral anomalies and no spondylolysis or spondylolisthesis affecting the lumbosacral junction. The hips were normal with congruent joints and well-contained femoral heads. The Risser grade was 1 indicating that the patient had a significant amount of remaining skeletal growth and was therefore at high risk for scoliosis deterioration.

Due to the severity of her scoliosis, a decision was made to proceed with surgical correction. In the presence of the underlying condition, a preoperative assessment was organized and this included a repeat head and spinal MRI, as well as cardiac, respiratory, anaesthetic, and neurology reviews.

**Figure 1:**
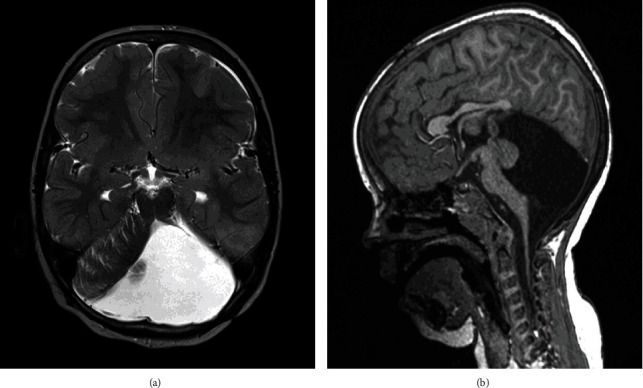
Axial T2 (a) and sagittal T1 (b) MRI scans show hypoplasia of the cerebellar vermis with cephalad rotation of the vermian remnant, cystic dilatation of the 4th ventricle extending posteriorly, anterior displacement of the cerebellar hemispheres, and enlargement of the posterior fossa. The torcula lies higher than the level of the lambdoid suture due to the abnormally high tentorium which is opposite of normal.

The MRI of the head and spine demonstrated hypoplasia of the cerebellar vermis and cystic dilatation of the 4th ventricle (Figure 1) and no vertebral, intraspinal, or paraspinal abnormalities. The cardiology assessment included an ECG and echocardiogram and found a structurally normal heart and no anomalies. Chest radiographs and sleep studies returned normal results and excluded apnoeic episodes. However, preoperative spirometry recorded FEV1 56% and FVC 56% predicted identifying a restrictive lung disease. A neurology review was undertaken and confirmed the absence of neurological abnormalities. No intervention was recommended in regard to the intracranial anomalies. The anaesthetic evaluation did not demonstrate any significant airway pathology that could complicate intubation and confirmed the patient's fitness to undergo scoliosis surgery. Blood test results including full blood count, urea, electrolytes, liver function tests, C-reactive protein, and coagulation screen were within normal limits.

**Figure 2:**
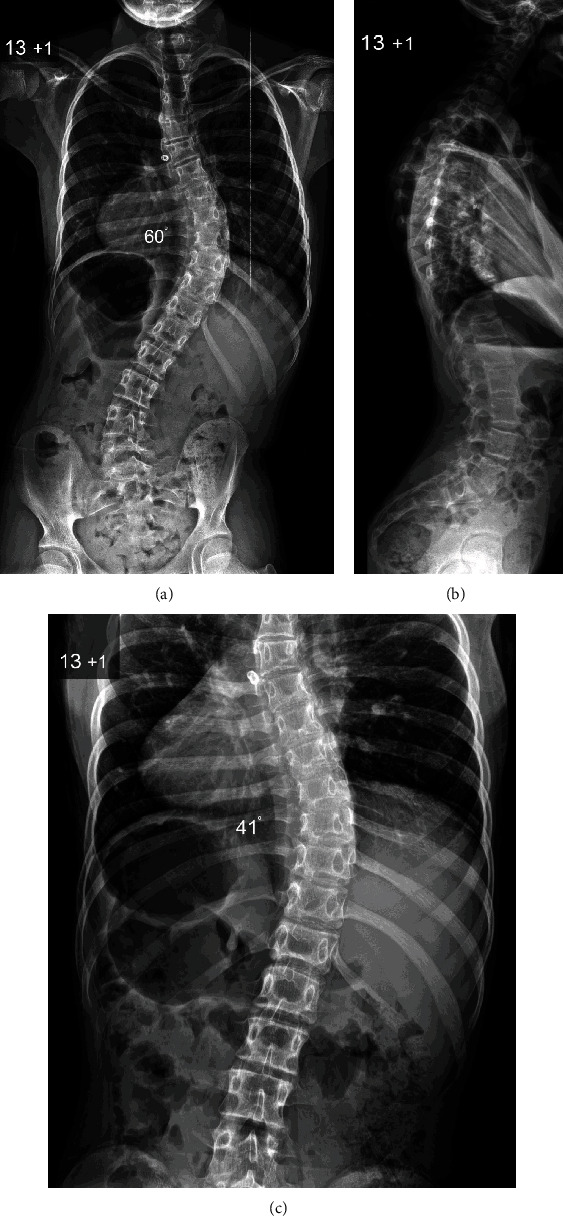
Preoperative posteroanterior (a) and lateral (b) radiographs of the spine show a severe right thoracic scoliosis producing thoracic translocation and spinal decompensation to the right with marked waistline asymmetry. Note that the global sagittal balance of the spine is retained as thoracic kyphosis and lumbar lordosis are compensated. However, the cervical spine is in a mildly kyphotic position. A supine X-ray was obtained with longitudinal traction (c) as part of the preoperative assessment, and this demonstrates that the thoracic curve retains mild flexibility.

Surgery was performed 4 months after initial presentation, when the thoracic scoliosis measured 60° (Figure 2). Thoracic kyphosis was normal, but there was increased lumbar lordosis associated with anterior pelvic tilt and a near horizontal sacrum. The patient underwent a posterior spinal fusion extending from T2 to L3 with pedicle hook, screw, and rod instrumentation and a combination of locally harvested autologous and allograft bone. The spinal fusion extended distally to the first lumbar stable vertebra (L3) and proximally to T2 in order to restore segmental and global sagittal balance of the spine and reduce the risk of proximal junctional kyphosis. The spine was exposed subperiosteally to the tips of the transverse processes and extensive facetectomies were performed in order to increase flexibility of the curve. No congenital vertebral abnormalities were identified. A bilateral hook/screw and rod construct was used, and this was secured to the spine by a combination of bilateral pedicle hooks with 20 mm locking screws extending from T2 to T4, as well as pedicle screws placed on the right side from T8 to L1 and bilaterally across L2 to L3. The deformity was corrected using apical segmental translation and a cantilever manoeuvre on the convex side, as well as proximal/distal distraction/compression of the construct. This was followed by extensive decortication of the posterior elements and onlay of autologous bone graft harvested from the spinous/transverse processes supplemented by allograft bone in order to achieve an intertransverse/interfacetal/interlaminar fusion. Intraoperative spinal cord monitoring was performed throughout the surgery recording cortical/cervical somatosensory (SSEP) and transcranial electrical motor (MEP) evoked potentials, as well as EMGs with no abnormalities detected.

Blood loss during surgery was 240 ml (11% estimated blood volume), and the patient did not require transfusion. Postoperatively, she was transferred to the high-dependency unit (HDU) where she remained for one day. She did not require oxygen support or nutritional supplementation through nasogastric/nasojejunal tubes. She had no neurological abnormalities and mobilised with the support of physiotherapists in the first 2 days after surgery. She did not require brace support. The patient was discharged 8 days after the procedure with radiographic evidence of good scoliosis correction and an adequate coronal and sagittal spinal balance.

**Figure 3:**
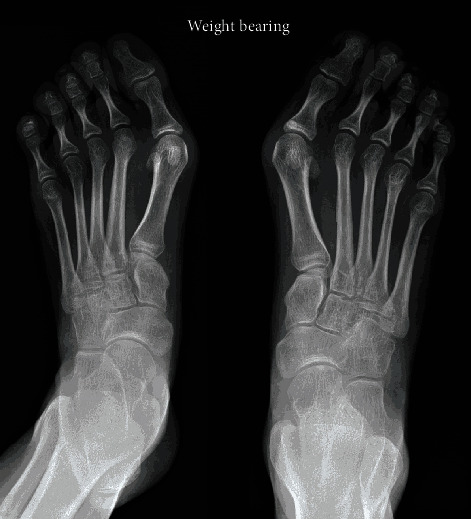
Bilateral hallux valgus which required surgical treatment after scoliosis surgery.

**Figure 4:**
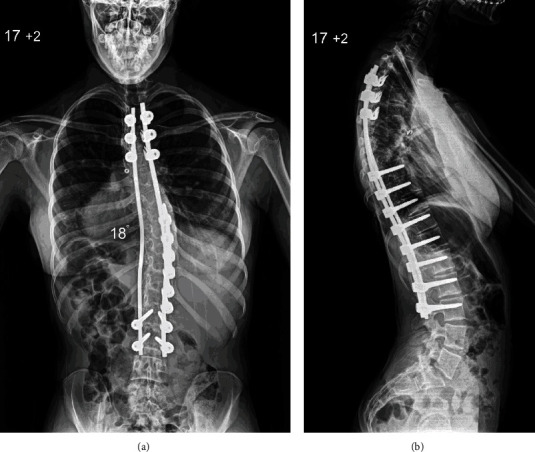
Posteroanterior (a) and lateral (b) radiographs of the spine at skeletal maturity show excellent scoliosis correction and a balanced spine in the coronal and sagittal planes.

The patient was reviewed closely in the months following surgery, and she returned to her normal level of activities with no complaints of her back. At 9 months postoperatively, she was referred for surgical treatment of bilateral hallux valgus (Figure 3). During her remaining skeletal growth, she was under the care of dieticians to optimise her body weight. At latest follow-up 4 years after surgery, the patient had completed her spinal development and she was happy with the surgical outcome. Spinal radiographs showed excellent deformity correction, which was maintained at follow-up with the instrumentation in good position and no evidence of pseudarthrosis (Figure 4).

## 3. Discussion

DWS is a rare neurological disorder that predominately affects the posterior cranial fossa as a result of a defect in embryonic development which occurs at an early stage. This embryonic defect produces a spectrum of congenital anomalies involving the 4th ventricle and cerebellum that affect brain development and are associated with the Dandy-Walker complex. The disorder was first described by Sutton in 1887 [6], further characterised by Dandy in 1914 [7], and refined by Walker in 1942 [8]; in 1954, Breda formally coined the collection of neurological abnormalities as the Dandy-Walker Syndrome [9]. The malformation affects around 1 in 25,000 live births and has female predominance [5, 10–12]. Patients with DWS have a number of brain anomalies such as hydrocephalus (70-90%), occipital encephalocele (16%), and partial or complete agenesis of corpus callosum (30%). A number of extracranial anomalies are associated with DWS affecting primarily the cardiac (54%) and with lesser frequency the gastrointestinal, craniofacial, and musculoskeletal systems [5, 10]. Our patient had a PDA and underwent surgical closure in early child life.

Children with DWS may have chromosomal abnormalities that are linked to associated syndromic disorders such as the Ritscher-Schinzel syndrome, but there is no isolated genetic anomaly identified that causes DWS. During embryonic growth, there is a regulated and chronological sequence of events in the formation of the posterior cranial fossa structures. The pathological process underlying the Dandy-Walker malformation is thought to arise from disruption of normal development of the membranous roof of the primitive 4th ventricle [10].

Antenatal diagnosis of DWS through evaluation of the posterior fossa structures is critical. Ultrasound imaging is the most common modality used in the prenatal setting to identify intracranial anomalies. The posterior cranial fossa can be sonographically evaluated with accuracy after 18 weeks gestation as prior to this embryonic stage there is a high false positive rate due to ongoing cerebellar vermis development [12]. A key feature for assessing growth of the cerebellum on the prenatal ultrasound is the transcerebellar diameter (TCD), with this measurement proving to be reliable in accurately differentiating the Dandy-Walker malformation from normal variants in the posterior fossa space.

Posterior fossa features suggestive of DWS on prenatal ultrasound can be further assessed with MRI, which similarly is not recommended until after 18 weeks gestation, although some authors suggest that MRI evaluation is only suitable after 24 weeks gestation [12]. Postnatal MRI studies are often performed to identify DWS and other intracranial anomalies in early neonatal life using standard protocols with sagittal T1 and axial T2 sequences. Our patient was diagnosed through postnatal MRI, and features indicative of DWS were seen including cerebellar hypoplasia, increased retrocerebellar fluid with enlargement of the posterior fossa, and the torcula lying higher than the level of the lamboid suture as a result of cystic dilation of the 4th ventricle (Figure 1). It is the elevation of the torcula that distinguishes normal variants of the posterior fossa from DWS [12].

Postnatally, patients with DWS have a characteristic phenotypic appearance. There are a number of craniofacial dysmorphic changes including strabismus, hypertelorism, slant of palpebral fissures, globular nose, cleft palate, poorly lobulated ears, large mouth with downturning corners, and high arched palate. Musculoskeletal abnormalities including small hands and feet, clinodactyly, and brachymesophalangy of the little fingers are often present [5]. Clinical examination can identify features indicative of raised intracranial pressure with children often having a bulging occiput and macrocephaly [13]. Our patient had the typical craniofacial dysmorphic features of DWS, which facilitated diagnosis at a young age.

Many children with DWS have ataxia and impaired coordination, which was present in our patient. Limb hypotonia is noted in young age, but over time, hypertonia and limb spasticity are frequently found [14]. Previous reports have demonstrated an increased prevalence of psychosis and endocrinological abnormalities such as hypoparathyroidism associated with DWS [15, 16]. Developmental delay can be associated with posterior fossa malformations, but this is primarily present in children with DWS associated with other syndromic or neurological abnormalities [4]. Our patient had isolated DWS with no underlying genetic condition. Her initial motor development was delayed possibly due to her prematurity, but after early child life, she had normal function which was appropriate for her age.

The development of scoliosis in patients with DWS is a relatively unknown phenomenon with only one previous report in the literature [5]. Our patient presented with a right thoracic scoliosis, which was noted in early adolescence and was progressing at a stage of rapid pubertal growth. Surgical correction was therefore indicated, and a thorough preoperative assessment was conducted to ensure that she was in an optimal condition to undergo scoliosis correction and minimise the impact of associated comorbidities. Although our patient had a previous heart procedure in early child life, the preoperative cardiac assessment did not identify any structural or functional abnormality.

Our patient developed a right thoracic scoliosis without intraspinal anomalies, which resembles an adolescent idiopathic scoliotic deformity similar to the patient presented by Menon et al. [5]. However, she had normal thoracic kyphosis, which is different from typical adolescent idiopathic thoracic scoliosis as this is mainly associated with thoracic hypokyphosis or lordosis due to the axial/rotatory component of the deformity across the apical segments. In addition, our patient had increased distal lumbar lordosis and marked anterior pelvic tilt without lumbosacral spondylolysis/spondylolisthesis, which is more commonly seen in children with nonidiopathic (syndromic/neuropathic) scoliosis. The principles of surgical treatment, as well as her postoperative course, did not differ from adolescent patients with idiopathic scoliosis, and the final outcome was very satisfactory in regard to radiographic correction of the deformity, absence of back pain, and functional result at skeletal maturity.

## 4. Conclusion

Children with DWS can develop scoliosis with no clear causative relation. The deformity in our patient occurred during puberty and the adolescent growth spurt, which is the reason why it carried increased risk of deterioration. Scoliosis correction in the form of posterior spinal fusion was indicated in the presence of a progressive, severe deformity that produced spinal imbalance and mechanical back pain in order to improve cosmesis, maintain function, and prevent pulmonary complications. Associated comorbidities as part of underlying syndromic conditions have to be excluded, and a detailed preoperative assessment can exclude abnormalities that can affect the perioperative course. In the absence of major medical conditions, an uneventful postoperative course can be expected and a successful result can be achieved using surgical techniques similar to adolescent idiopathic scoliosis. An excellent surgical outcome can be maintained at follow-up to adult life and result in normal function and high patient satisfaction.
